# Analysis of postoperative effects of different semicircular canal surgical technique in patients with labyrinthine fistulas

**DOI:** 10.3389/fnins.2022.1032087

**Published:** 2022-11-03

**Authors:** Wei Meng, Mingjing Cai, Yanhui Gao, Hongbo Ji, Chuan Sun, Guangfei Li, Yanyan Wei, Yan Chen, Hui Ni, Min Yan, Shuangba He

**Affiliations:** ^1^Department of Otorhinolaryngology Head and Neck Surgery, Nanjing Tongren Hospital, School of Medicine, Southeast University, Nanjing, China; ^2^Department of Imaging, Nanjing Tongren Hospital, School of Medicine, Southeast University, Nanjing, China; ^3^Department of Operating Room, Nanjing Tongren Hospital, School of Medicine, Southeast University, Nanjing, China

**Keywords:** middle ear cholesteatoma, labyrinthine fistula, semicircular canal occlusion, analysis, hearing

## Abstract

**Objective:**

Different semicircular canal surgery techniques have been used to treat patients with labyrinthine fistulas caused by middle ear cholesteatoma. This study evaluated postoperative hearing and vestibular function after various semicircular canal surgeries.

**Materials and methods:**

In group 1, from January 2008 to December 2014, 29 patients with middle ear cholesteatoma complicated by labyrinthine fistulas were treated with surgery involving covering the fistulas with simple fascia. In group 2, from January 2015 to October 2021, 36 patients with middle ear cholesteatoma complicated by labyrinthine fistulas were included. Cholesteatomas on the surface of type I labyrinthine fistulas were cleaned using the “under water technique” and capped with a “sandwich” composed of fascia, bone meal, and fascia. Cholesteatomas on the surface of type II and III fistulas were cleaned using the “under water technique,” and the labyrinthine fistula was plugged with a “pie” composed of fascia, bone meal, and fascia, and then covered with bone wax.

**Results:**

Some patients with labyrinthine fistulas in group 1 exhibited symptoms of vertigo after surgery. In group 2 Patients with type II labyrinthine fistulas experienced short-term vertigo after semicircular canal occlusion, but no cases of vertigo were reported during long-term follow-up. “sandwich.” In patients with type II labyrinthine fistulas, the semicircular canal occlusion influenced postoperative hearing improvement. However, postoperative patient hearing was still superior to preoperative hearing.

**Conclusion:**

The surface of type I labyrinthine fistulas should be capped by a “sandwich” composed of fascia, bone meal, and fascia. Type II and III labyrinthine fistulas should be plugged with a “pie” composed of fascia, bone meal, and fascia, covered with bone wax.

## Introduction

Labyrinthine fistulas, also known as localized labyrinthitis or perilabyrinthitis, are a complication of chronic suppurative otitis media that is usually caused by cholesteatoma invading the osseous labyrinth and account for 4.9–12.7% of middle ear cholesteatomas (Rosito et al., 2019). The classification and intraoperative management of labyrinthine fistulas vary, and a clear standard is lacking. Whether the effect on inner ear function in labyrinthine fistulas with serious clinical lesions can be reduced when the cholesteatoma epithelium is completely removed remains controversial (Geerse et al., 2017; Lim et al., 2017). This controversy is due to the intraoperative opening of the labyrinth, which may lead to neurological hearing loss and the risk of further development of the remaining fistula, leading to suppurative labyrinthitis or cholesteatoma recurrence.

**FIGURE 1:**
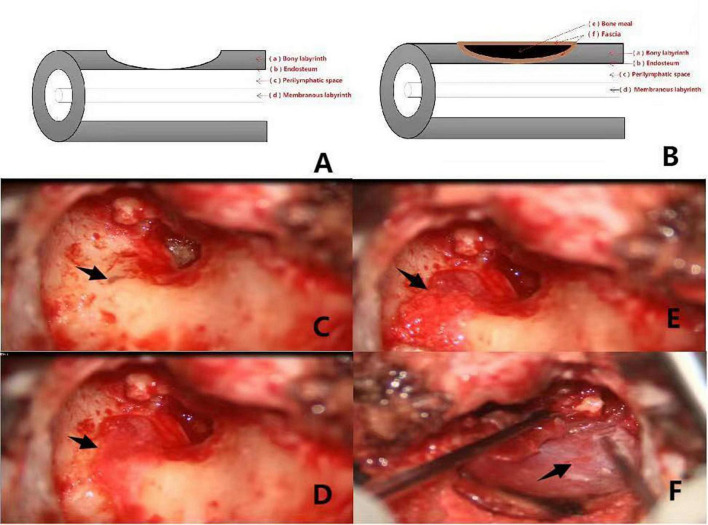
**(A,B)** Shows the preoperative and postoperative pattern diagram of a patient with type I labyrinteric fistula. The red arrows (a–f) respectively, represent the bony labyrinth, endosteum, perilymphatic space, membranous labyrinth, bone meal, fascia. Pictures **(C–F)** are intraoperative of a patient with type I labyrinthine fistula. **(C)**: Type I labyrinthine fistula with endosteum and cholesteatoma epithelium covered with surface (the black arrow). **(D)**: Fascia placed on the surface of the fistula (the black arrow). **(E)**: Surface of the fascia with clean bone meal (the black arrow). **(F)**: Fascia placed on the surface of the bone meal (the black arrow).

**FIGURE 2:**
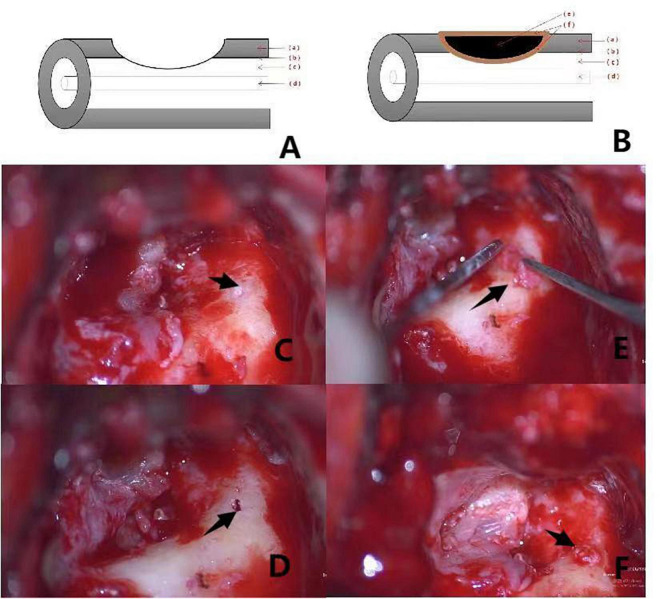
**(A,B)** Shows the preoperative and postoperative pattern diagram of a patient with type II labyrinteric fistula. The red arrows (a–f) respectively, represent the bony labyrinth, endosteum, perilymphatic space, membranous labyrinth, bone meal, fascia. Pictures **(C–F)** are intraoperative of a patient with type II labyrinthine fistula. **(C)**: Labyrinthine type II fistula with superficial cholesteatoma epithelium showing deep invagination within the external semicircular canals (the black arrow). **(D)**: Destruction of the endosteum is visible after “under water” cleaning of the cholesteatoma epithelium on the surface of the fistula (the black arrow). **(E)**: Placement of clean bone meal inside the fascia with a “pie” filling of the external semicircular canal (the black arrow). **(F)**: Filling the posterior surface of the semicircular canal by application of fascia is observed (the black arrow).

**FIGURE 3:**
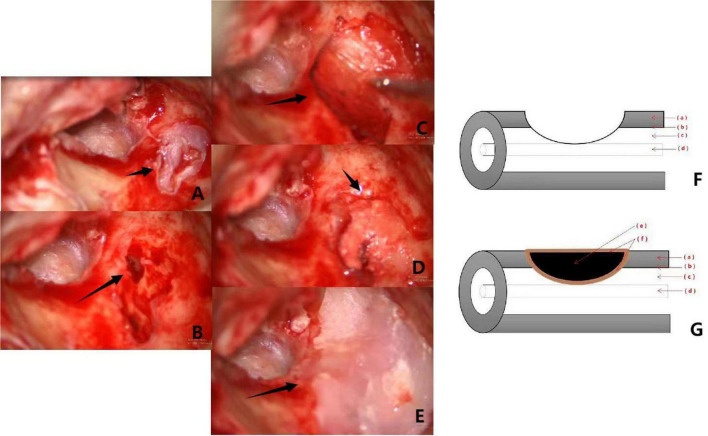
Pictures **(A–E)** are intraoperative of a patient with type III labyrinthine fistula. **(A)**: Type III labyrinth fistula with the superficial cholesteatoma epithelium located deep in the external semicircular canal (the black arrow). **(B)**: Cholesteatoma epithelium on the surface of the fistula (the black arrow). After the cholesteatoma epithelium is cleaned “under water,” the membrane labyrinth is destroyed. **(C)**: The fascia is placed on the surface of the fistula (the black arrow). **(D)**: Clean bone powder is placed on the fascia and the external semicircular canal is filled with a “pie” shape (the black arrow). **(E)**: The surface of the semicircular canal is covered with bone wax (the black arrow). **(F,G)** Show the preoperative and postoperative pattern diagram of a patient with type III labyrinteric fistula. The red arrows (a–f) respectively, represent the bony labyrinth, endosteum, perilymphatic space, membranous labyrinth, bone meal, fascia.

Among the various proposed classification criteria for labyrinthine fistulas, the widely used Dornhofer method divides fistulas, which are considered to be an erosion of the bony labyrinth with an intact endosteum, into three types (Dornhoffer and Milewski, 1995). A type I fistula is defined as an erosion of the bony labyrinth with an intact endosteum. A type II fistula is a true fistula consisting of an open perilymphatic space. A type III fistula consists of an open perilymphatic space with concomitant involvement or destruction of the underlying membranous labyrinth. This system is schematically represented in Figures 1A, 2A, 3F.

The latest classification, described by Quaranta et al. (2009), divides fistulas into six stages based on computed tomography and intraoperative findings. This staging system considers fistula size and depth.

Based on the convenience of the proposed analysis, we applied Dornhoffer and Milewski (1995) classification method in the present study.

According to the summary of surgical treatment techniques for superior semicircular canal dehiscence syndrome by previous scholars, the most commonly used surgical techniques for local bone defects in the superior semicircular canal involve application of various materials (such as bone, fascia, fat, and bone wax) (Mikulec et al., 2005; Ossen et al., 2017; Nguyen et al., 2018). The techniques were the plugging, resurfacing or capping. Studies showed that the success rate of occlusion was high (Mikulec et al., 2005). However, the occlusion technique is invasive and increases the risk of sensorineural hearing loss (Ziylan et al., 2017). Moreover, the type of reconstruction material may also affect the long-term results. Previous reports showed that autologous bone produced optimal results with less tissue inflammation. Conversely, fat, fascia, and muscle resulted in the lowest success rate (Kwok et al., 2019). In this study, we used some of the techniques described above to treat patients with labyrinthine fistulas caused by middle ear cholesteatoma. Our analysis included various combinations of autologous bone meal and fascia that were applied to repair bone defects in patients with different types of labyrinthine fistulas. In addition, postsurgical hearing and vestibular function were monitored.

## Materials and methods

We retrospectively analyzed the clinical data of group 1 and group 2 patients with middle ear cholesteatoma complicated by a labyrinthine fistula (Table 1).

**TABLE 1:** Characteristics of participants in groups 1 and 2.

Variable	Group	
	
	Group 1	Group 2
Number	29	36
Age (years)	51 ± 17.0	49 ± 1.33
**Sex**		
	Male 15	Male 19
	Female 14	Female 17
Side	Right 19	Right 20
	Left 10	Left 16
Type (Dornhoffer)	I 21	I 23
	II 8	II 12
	III 0	III 1
**Presentation**		
	With vertigo 5	With vertigo 9
	Without vertigo 24	Without vertigo 27

### Grouping

Group 1 included 29 patients with middle ear cholesteatoma complicated by labyrinthine fistulas that were treated between January 2008 and December 2014 (type I, *n* = 21; type II, *n* = 8; type III, *n* = 0).

Group 2 included 36 patients with middle ear cholesteatoma complicated by labyrinthine fistulas that were treated between January 2015 and October 2021 (type I, *n* = 23; type II, *n* = 12; and type III, *n* = 1).

### Surgical methods

Open radical mastoidectomy was performed to remove the cholesteatoma and expose the external semicircular canal, facial nerve, and auditory ossicle for all patients. The cholesteatoma at the labyrinthine fistula was temporarily retained and the entire intraoperative cavity was repeatedly rinsed with 1% adrenaline and dexamethasone saline. Dexamethasone was then injected into the operative cavity and the cholesteatoma epithelium at the labyrinthine fistula was removed “under water.” “under water” technology is throughout this step, saline containing corticosteroids was continuously superfused on to the area of surgery, creating a complete protective cover over the fistula and avoiding leakage of the perilymph, so that the removal of matrix and perimatrix was performed “under water” (Thangavelu et al., 2022).

#### Group 1

Surgical methods included open mastoidectomy, tympanoplasty, and facial nerve exploration. Intraoperatively, the researchers covered the labyrinthine fistula with simple fascia only, administered a dexamethasone injection, and proceeded with the operation.

#### Group 2

Type I fistulas were treated using open mastoidectomy + tympanoplasty + facial nerve exploration. Cholesteatomas on the surface of the labyrinthine fistula were cleaned using the “under water technique” and capped with a “sandwich” composed of fascia, bone meal, and fascia. Type II and III fistulas were treated with open mastoidectomy + tympanoplasty + facial nerve decompression + semicircular canal occlusion. Cholesteatomas on the surface of the labyrinthine fistula were cleaned using the “under water technique” and the fistula was plugged with a “pie” composed of fascia, bone meal, and fascia and then covered with bone wax. Hearing reconstruction was subsequently performed.

In one patient with a type III labyrinthine fistula and extremely severe preoperative mixed hearing loss, auditory ossicular chain reconstruction was not performed simultaneously.

All labyrinthine fistulas were located in the external semicircular canal.

### Images

Surgical images and pattern diagram of type I, II, and III labyrinthine fistulas in group 2 are shown in Figures 1–3.

### Postoperative treatment (7 days)

#### Anti-inflammatory therapy

Third-generation cephalosporins were administered intravenously for 7 days.

#### Anti-vertigo therapy

Betahistine tablets (1–2 tablets three times daily) were administered to patients with postoperative vertigo symptoms.

#### Hormone therapy

Daily hormone use was prescribed.

#### Nutritional neurotherapy

Ginaton (intravenous infusion) and mecobalamin (intravenous injection) were prescribed.

#### Daily observation

General patient conditions were observed, and bedside examinations for nystagmus were performed.

#### Postoperative checks

The bandage, the auricular and the incision of postauricular were observed at 3 days postoperatively, and once patients were able to sit independently, vestibular rehabilitation training was initiated.

### Statistical methods

Statistical analysis was performed using SPSS software (SPSS version 19.0; Wilcoxon signed-rank test). Differences were considered significant at *P* ≤ 0.05.

## Results

### Postoperative follow-up of middle-ear cholesteatoma patients with labyrinthine fistulas

A total of 65 patients with middle ear cholesteatoma and labyrinthine fistulas were followed up between January 2008 and October 2021, including 29 and 36 patients in groups 1 and 2, respectively. The patients were continuously followed up postoperatively. In group 1, middle ear cholesteatoma recurred in 2 of the 21 patients with type I labyrinthine fistulas.

### Comparisons of pre- and postoperative examinations

#### Pre- and postoperative hearing comparisons

##### Preoperative hearing comparisons

Bone conduction (BC) and air conduction (AC) thresholds were measured at frequencies of 250, 500, 1000, 2000, 4000, and 8000 Hz in the pure tone audiogram.

The average preoperative BC in patients with type I labyrinthine fistulas in groups 1 and 2 were 32.33 ± 0.48dB and 33.25 ± 0.72dB (*t* = 0.419, *P* ≥ 0.05) (Table 2).

**TABLE 2:** The average preoperative BC in patients with type I labyrinthine fistulas in group 1 and 2.

Frequencies	0.25 KHz	0.5 KHz	1 KHz	2 KHz	4 KHz	8 KHz
Group1 (dB)	35.29 ± 0.56	35.37 ± 0.52	30.33 ± 0.47	25.33 ± 0.72	30.29 ± 0.38	37.37 ± 0.23
Group 2 (dB)	35.00 ± 1.20	30.50 ± 0.39	30.75 ± 0.55	30.50 ± 0.84	35.25 ± 0.46	37.50 ± 0.88

The average preoperative BC: group 1 (dB) 32.33 ± 0.48; group 2 (dB) 33.25 ± 0.72. *t* = 0.419, *P* ≥ 0.05.

The average preoperative BC in patients with type II labyrinthine fistulas in groups 1 and 2 were 38.45 ± 0.35dB and 37.75 ± 0.35dB, (*t* = 0.207, *P* ≥ 0.05) (Table 3).

**TABLE 3:** The average preoperative BC in patients with type II labyrinthine fistulas in group 1 and 2.

Frequencies	0.25 KHz	0.5 KHz	1 KHz	2 KHz	4 KHz	8 KHz
Group 1 (dB)	40.66 ± 0.21	40.25 ± 0.10	40.55 ± 0.75	33.64 ± 0.62	35.25 ± 0.31	40.35 ± 0.11
Group 2 (dB)	40.51 ± 0.31	40.00 ± 0.11	40 ± 0.85	35.50 ± 0.52	35.50 ± 0.10	35.00 ± 0.21

The average preoperative BC: group 1 (dB) 38.45 ± 0.35; group 2 (dB) 37.75 ± 0.35. *t* = 0.207, *P* ≥ 0.05.

The average preoperative differences in air-bone (A-B) gap of patients with type I labyrinthine fistulas in groups 1 and 2 were 27.45 ± 0.72dB and 28.50 ± 0.45dB (*t* = 0.341, *P* ≥ 0.05) (Table 4).

**TABLE 4:** The average preoperative differences in A-B gap of patients with type I labyrinthine fistulas in groups 1 and 2.

Frequencies	0.25 KHz	0.5 KHz	1 KHz	2 KHz	4 KHz	8 KHz
Group 1 (dB)	30.55 ± 1.1	35.25 ± 0.91	20.66 ± 0.81	20.66 ± 0.51	22.33 ± 0.32	35.25 ± 0.67
Group 2 (dB)	35.25 ± 0.55	35.25 ± 0.67	20.65 ± 0.25	20.55 ± 0.45	24.05 ± 0.13	35.25 ± 0.65

The average preoperative differences in A-B gap: group 1 (dB) 27.45 ± 0.72; group 2 (dB) 28.50 ± 0.45. *t* = 0.341, *P* ≥ 0.05.

The average preoperative differences in A-B gap of patients with type II labyrinthine fistulas in groups 1 and 2 were 37.75 ± 0.24dB and 38.33 ± 0.35dB (*t* = 0.576, *P* ≥ 0.05) (Table 5).

**TABLE 5:** The average preoperative differences in A-B gap of patients with type II labyrinthine fistulas in groups 1 and 2.

Frequencies	0.25 KHz	0.5 KHz	1 KHz	2 KHz	4 KHz	8 KHz
Group 1 (dB)	40.50 ± 0.11	40.50 ± 0.21	30.25 ± 0.35	35.75 ± 0.55	35.5 ± 0.10	44.00 ± 0.12
Group 2 (dB)	40.29 ± 0.41	40.37 ± 0.32	35.33 ± 0.21	35.33 ± 0.55	35.29 ± 0.51	43.37 ± 0.10

The average preoperative differences in A-B gap: group 1 (dB) 37.75 ± 0.24; group 2 (dB) 38.33 ± 0.35. *t* = 0.576, *P* ≥ 0.05.

The average preoperative A-B gap of patients with type I and type II labyrinthine fistulas was not significantly different between groups 1 and 2.

##### Postoperative hearing comparison

In patients with type I labyrinthine fistulas, the average postoperative A-B gap of group 1 was not significantly different from that of group 2 (17.04 ± 0.65dB and 16.54 ± 0.38dB, *t* = 0.421, *P* ≥ 0.05) (Table 6).

**TABLE 6:** The average postoperative differences in A-B gap of patients with type I labyrinthine fistulas in group 1 and group 2.

Frequencies	0.25 KHz	0.5 KHz	1 KHz	2 KHz	4 KHz	8 KHz
Group 1 (dB)	15.25 ± 0.78	15.33 ± 0.55	15.75 ± 0.25	15.33 ± 0.97	20.29 ± 0.25	20.29 ± 1.1
Group 2 (dB)	15.25 ± 0.41	15.75 ± 0.21	14.75 ± 0.32	14.66 ± 0.75	18.83 ± 0.11	20 ± 0.48

The average postoperative differences in A-B gap: group 1 (dB) 17.04 ± 0.65; group 2 (dB) 16.54 ± 0.38. *t* = 0.421, *P* ≥ 0.05.

The average A-B gap of patients with type I labyrinthine fistulas was not significantly different between groups 1 and 2.

The average postoperative A-B gap of patients with type II labyrinthine fistulas was significantly different between groups 1 and 2 (22.25 ± 1.59dB and 28.33 ± 1.10dB, respectively, *t* = 3.772, *P* ≤ 0.05) (Table 7).

**TABLE 7:** The average postoperative differences in A-B gap of patients with type II labyrinthine fistulas in group 1 and group 2.

Frequencies	0.25 KHz	0.5 KHz	1 KHz	2 KHz	4 KHz	8 KHz
Group 1 (dB)	15.75 ± 3.2	20.50 ± 1.10	20.50 ± 2.50	20.75 ± 0.90	20.25 ± 1.21	35.75 ± 0.63
Group 2 (dB)	25.29 ± 2.50	25.29 ± 0.87	20.37 ± 1.20	25.33 ± 0.75	35.33 ± 0.2	38.37 ± 1.50

The average postoperative differences in A-B gap: group 1 (dB) 22.25 ± 1.59; group 2 (dB) 28.33 ± 1.10. *t* = 3.772, *P* ≤ 0.05.

The average A-B gap of patients with type II labyrinthine fistulas was significantly different between groups 1 and 2.

Patients with type III fistulas showed extremely severe sensorineural deafness on the affected side preoperatively that did not significantly improve postoperatively.

### Pre- and postoperative vestibular function examinations (caloric test, video head impulse test)

Caloric testing (at 30°C and 44°C) was performed in a completely dark room. The video head impulse test (vHIT) was performed while the patient sat on a chair and focused their eyes on a dot 1.5 m away on the opposite wall. After calibration, the examiner performed fast angular head movements in the planes of the semicircular canals (right horizontal–left horizontal, right superior–left posterior, and left superior–right posterior). This yielded the mean vestibular-oculo reflex (VOR) gains of all semicircular canals, calculated by the vHIT system as the ratio of the area under the curve of eye velocity to head velocity (from 60 ms before peak head acceleration to the last value of 0°/s as the head returns to rest). The diagnostic criteria for normal vestibular function are as follows: (1) caloric test: normal summation of caloric summed maximum slow-phase eye velocity (sMSPV) (°/s) is higher than 12°/s; (2) vHIT test: gain is normal when the VOR gain is between 0.8 and 1.2.

In patients with occasional preoperative vertigo, vestibular function was examined preoperatively, and at 1 week and 3 months postoperatively, and symptoms were followed up simultaneously.

#### Type I labyrinthine fistula patients

Type I patients in groups 1 and 2 showed no preoperative symptoms of vertigo.

#### Type II and III labyrinthine fistula patients

Group 1: Five of eight patients reported vertigo with attractors during outpatient treatment, whereas, Two of the five patients reported experiencing sudden vertigo preoperatively without an obvious cause. Postoperatively, eight of erght patients experienced vertigo with an attractor during outpatient treatment. A vestibular function examination (caloric test) was performed preoperatively in two of eight patients and the vestibular predominance of one of two patients was partial to the healthy side, whereas the vestibular function of the affected side was weak. The vestibular function of the other patient was normal. In addition, the vestibular function of the two patients did not change significantly at 1 week or 3 months postoperatively (Table 8).

**TABLE 8:** Pre- and postoperative vestibular function examinations of type II labyrinthine fistula patients in group 1.

	1	2
Pre-operative caloric sMSPV (°/s)	26	9
1 week postoperative caloric sMSPV (°/s)	25	10
3 months postoperative caloric sMSPV (°/s)	27	12

Group 2: Eight of the twelve patients experienced preoperative episodes of sudden vertigo without obvious causes. Obvious symptoms of vertigo occurred during the first 1 weeks postoperatively. Symptoms resolved after drug treatment and vestibular rehabilitation training, even with attractors, during outpatient treatment. The patients’ daily activity levels returned to normal, and no episodes of vertigo occurred during long-term follow-up.

**FIGURE 4:**
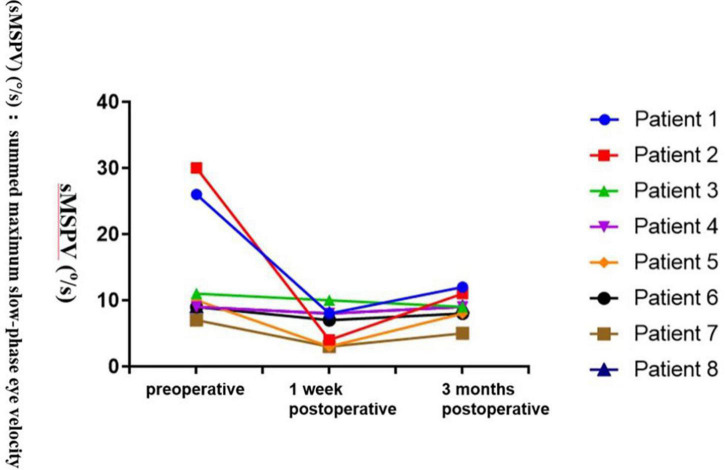
Caloric test results [summed maximum slow phase velocity of the eye, caloric sMSPV (°/s)] of the operated side of 8 patients with vertigo in group 2 before and after surgical plugging of external semicircular canal. Each patient is color coded. Vestibular function examinations performed at 1 week and 3 months postoperatively revealed that the affected side was weakened in all eight patients. The vestibular function of the patient was the worst one week after the operation, and gradually recovered in the later period, but it was not normal.

Preoperative vestibular function examinations (caloric test) revealed that the vestibular predominance of six of eight patients was partial to the healthy side, whereas the vestibular function of the affected side was weakened. The vHIT test on the affected side was positive, the gain was reduced, compensatory saccade waves appeared, and vestibular function was normal. Vestibular function examinations performed at 1 week and 3 months postoperatively revealed that the affected side was weakened in all eight patients (Figure 4 and Table 9).

**TABLE 9:** Pre- and postoperative vestibular function examinations of type II labyrinthine fistula patients in group 2.

	1	2	3	4	5	6	7	8
Preoperative ESCC VOR gain	0.85	1.0	0.35	0.42	0.51	0.36	0.30	0.56
1 week postoperative ESCC VOR gain	0.30	0.51	0.2	0.31	0.26	0.12	0.12	0.40
3 months postoperative LSCC VOR gain	0.56	0.62	0.33	0.40	0.46	0.39	0.29	0.50
Preoperative PSCC VOR gain	0.84	0.88	0.44	0.39	0.54	0.68	0.69	0.50
1 week postoperative PSCC VOR gain	0.87	0.78	0.30	0.5	0.55	0.58	0.70	0.49
3 months postoperative PSCC VOR gain	0.82	0.86	0.38	0.42	0.56	0.70	0.82	0.45
Preoperative ASCC VOR gain	0.83	0.84	0.57	0.68	0.50	0.68	0.54	0.67
1 week postoperative ASCC VOR gain	0.78	0.90	0.42	0.60	0.43	0.32	0.40	0.70
3 months postoperative ASCC VOR gain	0.85	0.87	0.50	0.56	0.58	0.70	0.36	0.70
Preoperative caloric sMSPV (°/s)	26	30	11	9	10	9	7	9
1 week postoperative caloric sMSPV (°/s)	8	4	10	8	3	7	3	8
3 months postoperative caloric sMSPV (°/s)	12	11	9	9	8	8	5	9

One patient with a type III labyrinthine fistula experienced dizziness for 4 years. Six months preoperatively, the vertigo worsened with occasional gait instability. Preoperative vestibular function examination (caloric test) revealed weakened vestibular function of the affected side and horizontal semicircular canal paresis, caloric sMSPV (°/s): 2, vHIT: The test on the affected side was positive, ESCC VOR gine was 0.35. Although the unstable gait and vertigo persisted, they gradually disappeared after the vestibular rehabilitation training after surgery. The vestibular function (caloric test) of the affected side was eased at 3 months compared to one week after surgery, caloric sMSPV (°/s): 4,vHIT: The test on the affected side was positive, ESCC VOR gine was 0.59.

### Imaging examinations: Pre- versus postoperative

**FIGURE 5:**
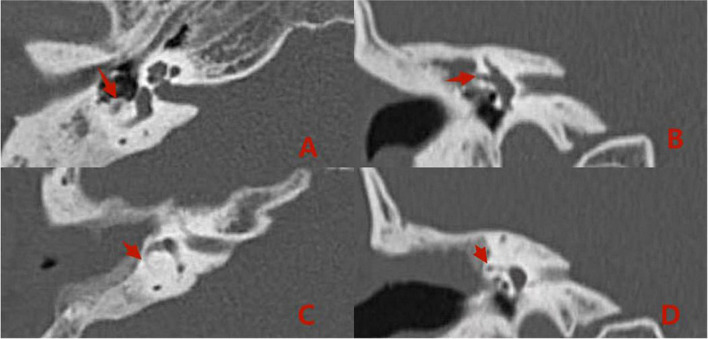
Computed tomography (CT) images acquired **(A,B)** preoperatively from a patient with type I labyrinthine fistula. Preoperative **(A,B)**: The right cholesteatoma is adjacent to bone resorption, the boundary with the external semicircular canal is unclear, and the shape of the external semicircular canal is acceptable (the red arrows). Postoperative **(C,D)**: The cholesteatoma has been removed, the shape of the lateral semicircular canal is acceptable, and intraoperative fillers are applied to fill the external semicircular canal (the red arrows).

Pre- and postoperative imaging examinations of patients with type I fistulas in the group 2 are shown in Figure 5.

**FIGURE 6:**
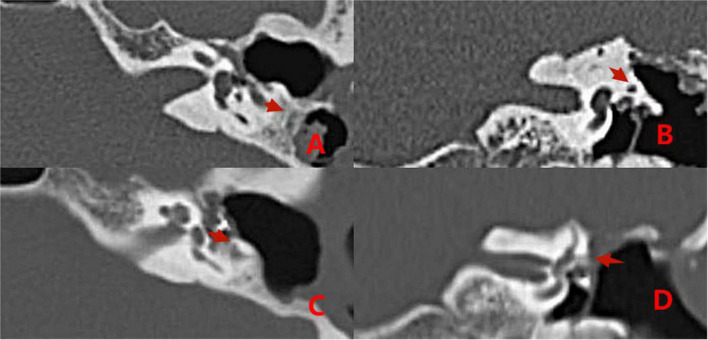
CT images acquired before and after surgery from a patient with type II labyrinthine fistula undergoing a second surgery. Preoperative **(A,B)**: The left cholesteatoma is adjacent to the bone resorption with a normal external semicircular canal shape (the red arrows). Postoperative **(C,D)**: The removal of cholesteatoma, the abnormal shape of the external semicircular canal, and the intraoperative fillers inside and adjacent to it (the red arrows).

Comparisons of preoperative and postoperative imaging examinations of patients in group 2 with type II fistulas are shown in Figure 6.

**FIGURE 7:**
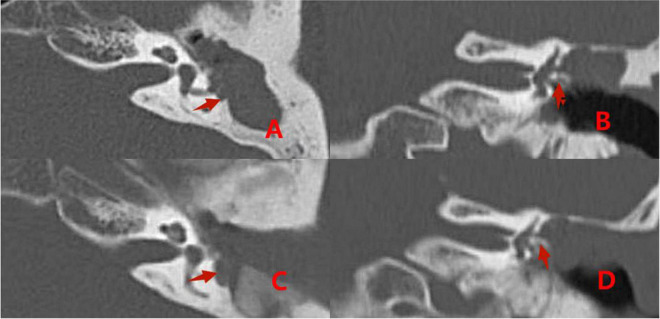
Pre- and postoperative CT images acquired from a patient with a type III labyrinthine fistula. Preoperative **(A,B)**: The left cholesteatoma is adjacent to the bone destruction (the red arrows). Postoperative **(C,D)**: The removal of the cholesteatoma, the abnormal shape of the external semicircular canal, and intraoperative fillers inside and adjacent to it (the red arrows).

Comparisons of preoperative and postoperative imaging findings in group 2 patients with type III fistulas are shown in Figure 7.

## Discussion

Labyrinthine fistulas are a common complication of middle ear cholesteatoma. Typically, treatment is difficult and several related factors influence the postoperative effects. For example, the rate of preoperative vertigo, degree of damage to the external semicircular canal, preoperative hearing, and vestibular function affect repeat operations and recovery of postoperative ear symptoms. Management of labyrinthine fistulas secondary to middle ear cholesteatoma remains controversial. Two methods have been described in the literature. One method advocates leaving the cholesteatoma epithelium to temporarily cover the fistula to avoid inducing further damage to the labyrinthine structure, as the opening of the labyrinth might further damage cochlear function. Another technique advocates the complete removal of cholesteatoma in the fistula area and sealing through the bone or cartilage, as residual cholesteatoma can progress with further bone resorption and destruction, causing further hearing loss; however, there is also a risk of intracranial complications (Djalilian et al., 2021). This study applied Dornhoffer and Milewski (1995) fistula classification and reviewed follow-ups of patients previously treated for labyrinthine fistulas caused by middle ear cholesteatoma to determine the ideal surgical treatment.

### Analysis of postoperative vertigo symptoms and vestibular function

Postoperative follow-up revealed that patients with type I or II labyrinthine fistulas in group 1 experienced vertigo after exposure to attractive stimulation. The researchers suspected that this was due to a lack of cortical bone protection for the labyrinthine fistulas after surgery. Patients with type I labyrinthine fistulas in group 2 had no symptoms of vertigo because the surface of the labyrinthine fistula had been capped intraoperatively with a “sandwich” of fascia, bone meal, and fascia.

In patients with type II labyrinthine fistulas that were plugged with fascia, bone meal, and bone wax, obvious vestibular symptoms occurred 1 week postoperatively due to intraoperative stimulation of the semicircular canal. In those cases, we observed a significant decrease in the patient’s vestibular functions 1 week after the operation due to provision of postoperative anti-vertigo drug therapy that was intended to effectively alleviate postoperative vestibular symptoms. Subsequently, no vertigo occurred during the long-term follow-up.

The follow-up of vestibular symptoms in type III patients showed that the vestibular symptoms persisted after the surgery, and they were relieved by drugs and vestibular rehabilitation training. No cases of vertigo occurred during the long-term follow-up.

Similar to results from previous studies, plugging of a semicircular canal could affect both vestibular function and hearing. After the initial deterioration, most patients recovered during the follow-up period. However, vestibular function loss can persist (Geerse et al., 2017; Misale et al., 2019).

### Analysis of postoperative hearing changes

The preoperative BC did not differ significantly between groups 1 and 2.

The average A-B gap did not differ significantly between groups 1 and 2. Specifically, in patients with type I labyrinthine fistulas, the removal of cholesteatoma on the labyrinthine surface of the fistula and the “sandwich” capping of two layers of fascia and bone meal did not affect the postoperative hearing improvement.

We found a significant difference in the average A-B gap between groups 1 and 2 due to the destruction of the membrane labyrinth caused by plugging the semicircular canal with fascia and bone meal during the operation of type II labyrinthine fistulas. This approach influenced postoperative hearing improvement. However, postoperative patient hearing was still superior to preoperative hearing.

Patients with type III fistulas showed extremely severe sensorineural deafness preoperatively with no significant postoperative changes.

Similar to previous studies, we found that hearing loss could persist after surgery (Jiang et al., 2022; Kontorinis and Thachil, 2022; Stultiens et al., 2022).

### Importance of complete intraoperative cholesteatoma removal

Complete cholesteatoma removal is the main purpose of surgery in patients with middle ear cholesteatomas. In particular, cholesteatoma scurf on the fistula surface requires complete removal to prevent postoperative recurrence or further spread of the inner ear infection. Previous studies (Wiatr et al., 2015; Bo et al., 2016; Rah et al., 2018) showed no significant effects on the preservation of BC hearing in the three types of labyrinthine fistulas; thus, removal of the cholesteatoma matrix had no significant effect on postoperative BC hearing in patients with middle ear cholesteatoma accompanied by labyrinthine fistulas. The follow-up of patients with labyrinthine fistulas in the present study showed that the use of appropriate surgical methods could preserve partial hearing in patients who underwent complete cholesteatoma removal. In patients with type I labyrinthine fistulas, the endosteum was not destroyed by the cholesteatoma; therefore, after removal of the epithelium of cholesteatoma on the surface of the labyrinthine fistula, the surface was capped by the “sandwich” method and the hearing was reconstructed simultaneously. In patients with type II labyrinthine fistulas, due to bone labyrinth destruction, we operated cautiously and simultaneously plugged the semicircular canal with a “pie” when removing the cholesteatoma epithelium. In this study, after the reconstruction, postoperative hearing was affected by operation of the semicircular canal. However, hearing examinations showed that postoperative patient hearing was still superior to preoperative hearing. Consequently, the researchers chose surgery involving complete cholesteatoma removal for middle ear cholesteatoma patients with labyrinthine fistulas.

### Significance of pre- and postoperative hormone use

In this study, the surgeon used the “under water” surgical method after clearing the cholesteatoma (Yamauchi et al., 2014; Hassannia et al., 2019; Creighton et al., 2021; Thangavelu et al., 2022). Specifically, before operating on the labyrinthine fistula, the researchers injected dexamethasone into the operation area. The epithelium of the cholesteatoma on the surface of the labyrinthine fistula was cleaned, and the corresponding semicircular canal operation was performed according to the labyrinthine fistula classification. This approach provided the following two advantages: protecting the inner ear from accidental gas interference at the liquid level, and preventing accidental perilymph inhalation and membrane labyrinth destruction (Zhang et al., 2021; Kawamura et al., 2022). Postoperatively, intravenous corticosteroids were continued to stabilize inner ear function and relieve possible symptoms of vertigo.

## Conclusion

Our analysis of the various management methods for semicircular canals in patients with different labyrinthine fistulas caused by middle ear cholesteatoma led to the following conclusions:

In patients with type I labyrinthine fistulas, the surface of the fistula should be capped with a “sandwich” composed of fascia, bone meal, and fascia. Our results showed that hearing improved postoperatively, and no vertigo occurred during the long-term follow-up.

In patients with type II and III labyrinthine fistulas, the fistulas should be plugged with a “pie” composed of fascia, bone meal, and fascia and then covered with bone wax. The semicircular canal occlusion influenced postoperative hearing improvement. However, postoperative patient hearing was still superior to preoperative hearing. No vertigo episodes occurred during long-term follow-up.

## Data availability statement

The original contributions presented in this study are included in the article/supplementary material, further inquiries can be directed to the corresponding author.

## Ethics statement

The studies involving human participants were reviewed and approved by Ethics Committee of Nanjing Tongren Hospital. The patients/participants provided their written informed consent to participate in this study. Written informed consent was obtained from the individual(s) for the publication of any potentially identifiable images or data included in this article.

## Author contributions

WM and MC: writing—original draft preparation. YG, HJ, and YW: methodology and data curation. CS and GL: find the relevant knowledge. YC, HN, and MY: provide the clinical data. SH: writing—review and editing. All authors contributed to the article and approved the submitted version.
